# Guidelines for treatment naming in radiation oncology

**DOI:** 10.1120/jacmp.v17i2.5953

**Published:** 2015-11-07

**Authors:** Travis R. Denton, Lisa B. E. Shields, Michael Hahl, Casey Maudlin, Mark Bassett, Aaron C. Spalding

**Affiliations:** ^1^ The Norton Cancer Institute Radiation Center Louisville KY; ^2^ Associates in Medical Physics, LLC Greenbelt MD; ^3^ Norton Neuroscience Institute Louisville KY; ^4^ The Brain Tumor Center Norton Healthcare Louisville KY USA

**Keywords:** radiation therapy, naming convention, nomenclature, record and verify, quality management, quality improvement, standardizing

## Abstract

Safety concerns may arise from a lack of standardization and ambiguity during the treatment planning and delivery process in radiation therapy. A standardized target and organ‐at‐risk naming convention in radiation therapy was developed by a task force comprised of several Radiation Oncology Societies. We present a nested‐survey approach in a community setting to determine the methodology for radiation oncology departments to standardize their practice. Our Institution's continuous quality improvement (CQI) committee recognized that, due to growth from one to three centers, significant variability existed within plan parameters specific to patients’ treatment. A multidiscipline, multiclinical site consortium was established to create a guideline for standard naming. Input was gathered using anonymous, electronic surveys from physicians, physicists, dosimetrists, chief therapists, and nurse managers. Surveys consisted of several primary areas of interest: anatomical sites, course naming, treatment plan naming, and treatment field naming. Additional concepts included capitalization, specification of laterality, course naming in the event of multiple sites being treated within the same course of treatment, primary versus boost planning, the use of bolus, revisions for plans, image‐guidance field naming, forbidden characters, and standard units for commonly used physical quantities in radiation oncology practice. Guidelines for standard treatment naming were developed that could be readily adopted. This multidisciplinary study provides a clear, straightforward, and easily implemented protocol for the radiotherapy treatment process. Standard nomenclature facilitates the safe means of communication between team members in radiation oncology. The guidelines presented in this work serve as a model for radiation oncology clinics to standardize their practices.

PACS number(s): 87.56.bd, 87.56.Fc, 87.55.Qr, 87.55.‐x, 87.55.N‐, 87.55.T‐, 87.55.D‐

## I. INTRODUCTION

Radiation oncology relies on the principles of process improvement as a means of promoting quality and development in the delivery of the therapies involved in clinical practice. Standardization of workflow, processes, and personnel training requires integration of multiple technical and human components. Standard naming conventions are necessary for safe delivery of irradiation; however, they alone are not sufficient.^1^, ^2^, ^3^ Nomenclature, as defined for the radiation oncology environment, is the devising of terms for components of the radiation prescribing, simulation, planning, or treatment process. These terms should have a basis in logic and be reproducible outside of an individual or local group within the clinic.^(4)^ Standards used in health care have been found to significantly improve the accuracy of achieving the physician's intent of treatment, the effectiveness of communication between individual radiation oncology team members and software‐based platforms, and the quality of patient record‐keeping.^5^, ^6^, ^7^, ^8^, ^9^, ^10^, ^11^, ^12^

Inconsistencies or poorly chosen naming practices in a radiation oncology clinic may lead to confusion in the interpretation of important details, which may cause patient treatment errors or the misadministration of therapy.^13^, ^14^, ^15^, ^16^, ^17^ It has been reported in the literature that lack of naming standardization contributes to the incident of event reporting via mechanisms such as PSRS.^(18)^ Changes in practice must be based on a careful assessment of the needs of that change, as well as understanding of the causes that could lead to treatment errors.^(19)^ The PSRS in conjunction with CQI programs provide an excellent mechanism from which to evaluate, present, and drive valid changes in practice centered around improving the safety and efficacy of patient treatment.^20^, ^21^, ^22^ In general, naming conventions can help practitioners cope with the ever expanding levels of sophistication of modern treatment simulation, planning, and delivery systems, and increase the safety culture of a clinic.^7^, ^22^, ^23^, ^24^

There can be difficulties in universal adoption of naming conventions.^(25)^ The successful implementation of a convention requiring a change in practice in a clinic must overcome barriers such as cognitive, attitudinal, professional, practicality, and lack of organization.^19^, ^26^ The challenge is illustrated by noting the gap that may exist between research recommendations, clinical practice guidelines, and actual clinical practice in a clinic setting.^(19)^

We conducted this study to determine the methodology for radiation oncology departments to standardize their practice with external beam, brachytherapy, and unsealed sources. We present here our quantitative, iterative methodology for improved patient safety based on input from clinical and system quality staff.

## II. MATERIALS AND METHODS

The department in this study operates five linear accelerators and provides services for external beam radiotherapy, intensity‐modulated radiotherapy (IMRT), stereotactic radiosurgery, stereotactic body radiotherapy, high‐dose‐rate brachytherapy, low‐dose‐rate brachytherapy, Y‐90 radiopharmaceutical therapy, and I‐131 radiopharmaceutical therapy for three separate physical clinic sites operating under the umbrella of a single institution. The patient management system used intradepartmentally is Aria (Varian Medical Systems, Palo Alto, CA), while the interhospital department electronic medical record system is EPIC (Epic Systems Corporation, Madison, WI). The treatment planning systems utilized are the following: Eclipse (Varian Medical Systems), iPlan (Brainlab AG, Feldkirchen, Germany), BrachyVision (Varian Medical Systems), and VariSeed (Varian Medical Systems).

The institution's CQI committee recognized that due to growth from one to three centers, significant variability existed within plan parameters specific to patients’ treatments. The committee determined that an institution‐wide, multidisciplinary‐derived naming standard would help mitigate the potential risk for mistreatment that may result from ambiguously naming treatment parameters. The committee was charged with standardization of universal processes to reduce risk.

The committee commissioned an effort to provide a naming convention that could be implemented by all of the radiotherapy staff members. The group designated to compose the general guidelines in standard treatment naming presented in this study was formed on January 8, 2014. The report was implemented by the institution on February 1, 2015. The entire scope of the CQI is described in Table 1, while this manuscript focuses on Phase I of this table. The committee consisted of three committee members (Physicist/Dosimetrist, Radiation Therapy Technologist, and Radiation Oncologist) and one team leader (Director of Radiation Oncology Services). We gathered input using electronic and anonymous surveys disseminated on a regular basis with each survey addressing a different subcomponent of the scope of the project. The purpose was to encourage maximum participation by minimizing the time needed to complete each survey and to provide clear start and end dates for the timely completion of each survey.

**Table 1:** Global scope of the nomenclature standardization efforts

	*Radiation Oncology Nomenclature Scope*	
*Phase*	*Phase Title*	*Phase Components/Goals*
I	Treatment Planning Parameters	Anatomical Site, Treatment Course, Treatment Plan, Treatment Fields, Standard Units of Physical Quantities, Forbidden Characters
II	Document Naming	Consultation, Consent, Orders, Visits, End‐of‐Treatment, Follow Up
III	Treatment Activity Naming	Standardizing activity titles
IV	Billing	Providing clear billing guidelines
V	Treatment Care Path Template	For various treatment modalities (e.g. EBRT, IMRT, SBRT, HDR)

A system was created for naming standardization of the following: anatomical sites, treatment course, treatment prescription, treatment plan, and treatment field. Additional concepts were addressed including, the use of capitalization, specification of laterality, course naming in the event of multiple sites being treated within the same course of treatment, primary versus boost planning, the use of bolus, revisions for plans, image‐guidance field naming, forbidden characters, and standard units for commonly used physical quantities in radiation oncology practice. This formalization was made with the understanding of the limitations derived during the use of the patient management software employed in a typical radiation therapy environment and with the interests of risk mitigation at the forefront of the establishment of the nomenclature as used by all members of the radiation oncology team.

Each survey was available for one week and was followed immediately by the next survey. Designed to be able to be completed in approximately five minutes, each survey consisted of three to five questions consisting of multiple‐choice responses, free‐form comment fields, and ranking question types. A scoring system was used in which options receiving greater preference status in the responses were assigned greater scores. The scores were summed for each option and divided by the sum of the number of responses for that question. For each, if an option (considered against three other options) received one most preferred ranking, two second most preferred rankings, zero third preferred rankings, and three least preferred rankings for a question with six responses gathered, then the score would be:

(1) $$\begin{array}{l} Score=\frac{\left(1)(Value_{1})+(2)(Value_{2})+(0)(Value_{3})+(3)(Value_{3}\right)}{6}\\ \;\;\;\;=\frac{\left(1)(4\;\text{points})+(2)(3\;po\text{int}s)+(0)(2\;po\text{int}s)+(3)(1\;po\text{int}\right)}{6}=2.17 \end{array}$$

It was the intent of the committee to be able to take advantage of the expertise and experience of this extended group of professionals and address previously unconsidered issues that were pointed out during the course of collecting these surveys. The responses by the users were recorded anonymously, and the compiled results from the survey were not shared outside of the standardization committee prior to the release of the general guidelines for standard treatment naming for the radiation oncology department.

The committee compiled the input from these surveys to establish specific conventions. These conventions provided universal guidance for problematic naming ambiguities. By giving the team members a documentable forum for their input, the smaller composition group was able to ensure that the guidelines were produced in a finite and realistic time‐frame and that buy‐in for these guidelines could be maximized to promote a more successful adoption of the end result. Our goal with this effort was to raise awareness of how to achieve successful and meaningful change within a radiotherapy environment while considering the psychological impact of behavior modification, the effect of buy‐in, and effectively using constructive feedback.

Table 2 illustrates how the methodology was utilized to gather consensus. Step one asked the question “Please rank your preference regarding the specification ‘Right’”. Three choices were listed: 1) Rt, 2) RT, 3) R. The data were then compiled with a weighted average. Table 2 reflects the frequency of being ranked #1. In this example, “Rt” was used moving forward as the accepted consensus. Similar polling with ranked choices for other conventions such as course naming, treatment plan naming, and treatment field naming was used. The GTV, CTV, and PTV nomenclature reflects the minimum required terms that are accepted within our department to define consensus for how to name them.^(27)^ When multiple targets within a plan or sequential boost plans occur, additional clarification is needed.

The guidelines of the treatment naming standard presented in this study incorporated several critical features (Table 3). The flexibility requirement, mentioned above, is illustrated via the example that structure names for two hypothetical structures: “limb” and “extremity” could be meaningfully interpreted by a colleague to mean the same thing without causing confusion. The use of structure names such as “arm” and “limb” could cause confusion. Therefore, the convention should allow for a reasonable degree of flexibility, but not to the extent that it allows for naming confusion. This requirement for flexibility was integral in assuring the successful implementation of the convention as had been cited in other examples of efforts to implement changes in practice in a clinic setting.^(4)^ These requirements promoted confidence in adopting of the end result.^(28)^

**Table 2:** Methodology to gather consensus in treatment naming in radiation oncology

	*1*	*2*	*3*	*Total*	*Average Ranking*
Rt	10 (83.33%)	2 (16.67%)	0 (0.00%)	12	2.83
RT	1 (8.33%)	9 (75.00%)	2 (16.67%)	12	1.92
R	1 (8.33%)	1 (8.33%)	10 (83.33%)	12	1.25

**Table 3:** Features of a treatment naming standard

Realistically implementable^(34)^
Sufficiently brief, readable, and fonctional^(41)^
Inclusive of concrete and specific statements^(42)^
Adaptable and relevant to current practices^(17)^
Representative and embodying quality communication^(34)^
Mindful of resource implications^(43)^
Clinically flexible^(4)^
Able to be evaluated via retrospective audit post implementation^(44)^

## III. RESULTS

### A. Survey participation

**Figure 1:**
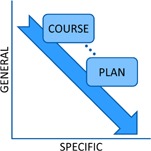
The general‐to‐specific philosophy of plan parameter naming as applied to radiation oncology treatments. Course naming can be generalized to a greater degree to allow for accurate and meaningful naming while not demanding impossible naming restrictions. At the plan level though, a greater deal of specificity is required.

**Figure 2:**
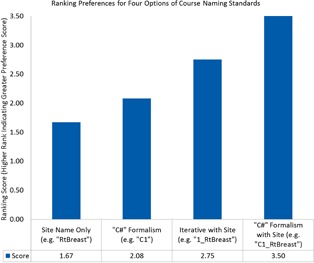
Ranking preferences for four options of course naming standards. This plot shows the ranking, sorted in ascending order, for four options presented during the intra‐departmental survey outreach. The greater scores are associated with the more preferred standards from the polling of the survey recipients within the department. The question prompt for this survey question was: “Please rank your preference regarding the following examples relevant to course naming (1 is your most preferred and 4 is your least preferred option).

**Figure 3:**
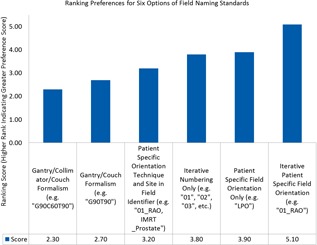
Ranking preferences for six options of field naming standards. This plot shows the ranking, sorted in ascending order, for six options presented during the intradepartmental survey outreach specific to treatment field naming standards. The greater scores are associated with the more preferred standards from the polling of the survey recipients within the department. The question prompt for this survey question was: “Please rank your preference regarding the following examples relevant to treatment field naming (1 is your most preferred and 4 is your least preferred option).

The committee solicited input by means of brief electronic surveys from all physicians, physicists, dosimetrists, chief therapists, and nurse managers. The completion rate was 73.0% (35 total responses received from 48 survey prompts divided amongst three surveys). These surveys and high completion rate allowed the iterative development of uniform naming standards, based on using more general terms for courses and more details for treatment plan parameters (Fig. 1). The surveys were designed with a limited number of choices for two reasons. First, this allowed quantitation of the results. Second, it facilitated adoption of the recommended choice. Figure 2 demonstrates the weighted responses from the survey used to obtain a consensus for naming a course of treatment. This clearly shows the department preference for including both the number, laterality, and site within a course designation. Figure 3 outlines the results with a larger survey demonstrating identification of the most critical elements of the fields used in a treatment plan to include number and orientation.

### B. General guidelines for standard treatment naming for a radiation oncology department

**Use of Capitalization**: In writing compound words in the treatment planning and delivery workflow, it is recommended that the user use capitalized words for the start of each word with no spacing between words. This can extend to abbreviations with the first letter in the abbreviation being capitalized and the following in lower‐case. For example, should the user wish to abbreviate the word “right” to a two letter abbreviation, the user would write: “Rt”. **Writing Laterality**: It is recommended that, if only one laterality is needed to be specified, the longer form of the abbreviation be used (for example, “RtLung” instead of “RLung”). But, if multiple lateralities need specification, then the shorter is allowable (e.g., “RPO”). The shorter form of abbreviation may be necessary in light of certain character limitations. If it is possible to include the entire name, then it is acceptable to do so, with the understanding that other steps of the treatment workflow may require abbreviation.

**Laterality with Anatomical Site**: When writing a laterality combined with an anatomical site, the laterality shall be used as a prefix to the structure name. For example, in consideration of a right lung, the naming shall be “RtLung” (not, “LungRt”).

#### B.1 Naming of anatomical sites

The formalism proposed here for the naming of anatomical sites may be extended into all of the radiotherapy clinical components related to treatment planning and delivery where an anatomical specification is appropriate (including course, prescription, treatment plan, and treatment field naming) and should remain consistent throughout the documentation for a radiation oncology treatment plan. A summary of these conventions is provided in Table 4.

**Table 4:** Summary guide for the general guidelines for standard treatment naming for a Radiation Oncology Clinic

*Naming Level*	*Naming Rules*	*Examples*
General Capitalization	Capitalize the first letter of each new word.	RightLung
	Capitalize the first letter in each word of the abbreviated phrase.	RAO; LtObl
Laterality	Use only approved laterality abbreviations.	RtLung; LtOpticNerve
	Specify laterality before structure name.	
Course	Use the “C#” formalism.	C1, C2, C3, etc.
	Specify the treatment objective following the “C#” formalism.	C2_LtLung
	Include all plans within the same course number if plans are initially planned to be delivered concurrently or in physician directed succession.	C1_RightBreast; C1_Rbreast_bst
	Include all plans within the same course number if plans treated share a planning CT set.	
	Include all plans within the same course number if the multiple plans constitute the intended treatment at the time of physician specification of the intents.	
	Begin a new course number if a new plan is unrelated to the original treatment or not part of the intended treatment regime of the original course	
	If multiple sites are being treated in the same course, list the common structure (if applicable).	C1_Brain
	If multiple sites in the same course do not share a common structure, the treatment objective should be named to reflect the intention of treating multiple sites.	C3_MultipleMets
Prescribe Treatment	Anatomical site must be clearly labeled. Laterality should be clearly labeled.	
Treatment Plan	Plan name should consist of a component that matches the physician's intent exactly.	
	If possible, the plan name should include a qualifier suffix to indicate treatment technique.	RtLung_IMRT; RtLung_VMAT
	For boost plans, a qualifier suffix should be added of: “_bst”.	RtBreast_bst
	No qualifier suffix is necessary for primary plans.	
	Plan revisions should follow automatic naming conventions if possible and should include annotation detailing the need for the revision.	RtLung:2; RtLung:3; etc.
	Multiple stage plans should include a suffix qualifier to the plan name indicating the specification of the stage.	Larynx_Quad1
	Field‐in‐field plan names shall include the suffix “FinF.	RtBreast_FinF
Treatment Fields	Iterating field numbers should be used.	01, 02, 03, etc.
	An anatomical‐specific laterality indicator should be used.	01_LPO
	The hyphen symbol should be reserved for and only used for indicating transition.	02_LPO‐RAO (dynamic gantry treatment field example)
Setup Fields	The following field names can be used to denote setup fields: AP_kV, Rt_kV, Lt_kV, and CBCT.	

##### B.1.1 Summary of anatomical site guidelines

An anatomical site:

- Should use capitalization for the start of each new word (including laterality specifications)
- Should be used consistently throughout the plan records (though abbreviations are acceptable when necessary)
- Should list laterality specifications written before the anatomical site

#### B.2 Course naming

Course naming is recommended to include both a course designation character in addition to a course number.

##### B.2.1 Iterative course naming

Patients may require multiple courses of treatment for typical radiation oncology clinics. For the purposes of these guidelines with regards to radiation oncology, a treatment course is defined to:

- Be a fixed number of regular radiation derived medical treatments
- Be predetermined and explicitly specified (in terms of both number of fractions, dose per fraction, and frequency for fractions) by the physician prior to the delivery of radiation treatment
- Have a clearly defined intended target(s) of treatment assigned to specific treatment plan(s)
- Include only the treatment plans that are predetermined to be delivered at the initiation of the treatment regime developed by the physician (including boost plans)
- Include multiple plans for a patient if those plans are intended to be delivered concurrently or in a consecutive scheduled succession
- Include multiple plans for a patient if those plans share a common CT simulation acquired prior to treatment delivery
- Include multiple plans for a patient if those plans constitute, as a whole, the intended care for a particular patient

As such, the following requirements are recommended upon the naming of courses:

- The course names shall have a clear chronological order.
- Adoption of standardized templates based on disease site has made course naming semi‐automated as the site is populated once a template is chosen.
- The course name should include a brief descriptor of the treatment objective (for example, defining the explicit treatment site).

In light of this definition and these requirements, course naming recommends that the standardization via use of the “C#” formalism with the addition of the anatomical site to be treated in the relevant course of treatment appended in the name. For example, for a hypothetical patient's first course of treatment for a right breast site, the course name would be: “C1_RtBreast”. For subsequent treatment courses, the iteration of the course number is recommended without this course number being restarted. Thus, if this hypothetical patient's second course of treatment were to include treatment to a left lung site, then their second course name should be entitled: “C2_LtLung”.

##### B.2.2 Course naming in the event of multiple sites within the same course

If multiple treatment plans are necessary for a patient's treatment, as predetermined by the physician prior to the onset of radiation therapy, these plans should be included in the same course of treatment. The following two sections describe how to address the naming of a course that includes multiple plans of treatment meeting the above criteria.

###### B.2.2.1 Sites are in a shared anatomical site

If multiple plans are necessary within the same course of treatment and are to be delivered within a shared anatomical site, then the shared anatomical site should be included in the course naming. For example, for a patient receiving two treatment plans to a left frontal and right parietal metastatic lesion, the course name should be written as: “C1_Brain” or some variation of this indicating that the course of treatment contains cranial treatment plans. As another example, consider a right breast treatment which contains both a primary and a boost treatment. The course name for this patient should be written as: “C1_RtBreast”.

###### B.2.2.2 Sites are not in a shared anatomical site

For treatment plans within the same course and not delivered to a common site, the treatment course name is recommended to include a reflection of the general intention to treat multiple sites with the specificity of the site reserved for the physician's intent and the treatment plans. For example, a patient receiving a treatment to metastatic bone sites in both their pelvis and left humerus should have a course written as: “C1_MultipleMets” with plan names of: “Pelvis” and “LtHumerus”.

##### B.3 Naming for treatment prescriptions

The requirements for physician's intent naming as recommended by these guidelines include the following:

- The anatomical site must be clearly stated.
- The name shall adhere to the specifications detailed in Results section B.1.
- Laterality (where appropriate) shall be clearly stated and adhere to the format recommended in Results section B.1.

##### B.4 Treatment plan naming

The requirements for treatment plan naming are as follows:

- The plan name should match the physician's intent exactly.
- If possible, a qualifier should be affixed to the plan name to indicate technique.
- The plan name used in the treatment planning system should match the plan name in the record and verify system.

For example, if two plans are generated for a right lung treatment site with the first attempting IMRT as a treatment technique and the other volumetric‐modulated arc therapy (VMAT), then the two plans could be written as: “RtLung_IMRT” and “RtLung_VMAT”, respectively.

###### B.4.1 Primary vs. boost plan naming

When naming boost plans, it is the recommendation of these guidelines that a suffix of “_bst” be added to the standard format for plan naming to indicate that this plan corresponds with the physician's intent for a boost plan. When naming primary plans in the same course, no additional suffix is necessary. Simply follow the guidelines in the preceding section. For example, for a patient receiving both a primary and a boost treatment plan for a right breast treatment, the plan names could be written as: “RtBreast” and “RtBreast_bst”, respectively.

###### B.4.2 Bolus in plan naming

For treatment plans utilizing bolus, the use of bolus should not be specified in the treatment plan name as recommended here in the interest of character limitations, possible ambiguity, and the risk for mistreatment if the user should fail to adhere to the naming convention. Rather, the use of bolus should be explicitly included in the physician's intent, setup notes, and any interlock or sign‐off fields at the treatment delivery workstation.

###### B.4.3 Plan revisions

When revisions to treatment plans are deemed necessary after the initiation of the treatment course, the default naming convention used in the treatment planning system is recommended to be sufficient. For example, in Varian's Aria environment, the original plan's name is reproduced but also appended with “:#” with the numerical value increasing with each additional plan revision (to “:2”, “:3”, etc.). Relying on this automation will reduce errors due to transcription mistakes and will help preserve the continuity of treatment in a sufficiently clear manner for the purposes of treatment audit. Furthermore, the original plan name will remain available to be matched against the physician's intent.

###### B.4.4 Multistage plan naming

For plans that require multiple stages of delivery, the plan name for each stage is recommended to have a qualifier indicating that intent. For example, for a treatment followed by a reduction in treatment volume, a subsequent plan could be appended with “_Reduce1” to indicate the first reduction needed, and so forth.

###### B.4.5 Plans involving merged fields

For certain linear accelerators and the designs of their treatment heads, it may become necessary to merge treatment fields with common gantry/couch/collimator parameters but needing a shift in the multileaf collimators to allow for a different collimation of the treatment field. For such merged treatment fields, the postmerged treatment plan is recommended to be appended with the suffix of “FinF” (field‐in‐field). For example, merging treatment fields for a left breast plan would result in a field name of “LtBreastFinF”.

##### B.5 Treatment field naming

Treatment field naming may be written with patient‐specific anatomical orientation and iterative indicators to relate efficient field ordering and provide additional differentiation between field names. To further promote delivery efficiency and patient throughput, the field numbering prefix can begin with “01” and increase iteratively throughout the available fields in the treatment plan. Multiple fields described by the same anatomical orientation can be differentiated by the prefix acting as a unique identifier (for example, two fields, each incident on a patient from a separate left‐posterior oblique orientation can be made distinct by naming the fields: 01_LPO and 02_LPO). Furthermore, this numbering can continue with additional plans delivered in the treatment course, and with additional plans required of future courses of radiotherapy.

###### B.5.1 Dynamic gantry treatment fields

For arc‐based treatment fields, it is the recommendation of these guidelines that a hyphen be used to denote gantry motion. Thus, if, during the delivery of the treatment field, the gantry were to rotate from a left‐posterior‐oblique (LPO) to a right‐anterior‐oblique (RAO) patient‐specific orientation, then the field can be entitled: “01_LPO‐RAO” with the hyphen indicating the transition of the field from its starting to the field conclusion position. It is furthermore proposed that the hyphen only be used to indicate a transition or a range. In all other cases, the underscore (”_”) symbol should be used.

###### B.5.2 Image‐based setup field naming

Image alignment field naming can also be defined using these guidelines. For such fields, it is recommended that both the imaging modality and the patient‐specific orientation to which the digitally reconstructed radiograph (if applicable) was derived be explicitly stated in the name. For example, various kilovoltage (kV) setup fields and a cone‐beam CT (CBCT) can be routinely named as follows: AP_kV, Rt_kV, Lt_kV, and CBCT.

##### B.6 Allowed and forbidden characters typically encountered in patient management systems

Computerized patient management and treatment planning systems inherently include limitations on the number and types of characters available. Importantly, the limitations are not uniform across software platforms. However, information embedded in the naming of different plan parameters needs to be able to cross multiple platforms along the process from simulation to treatment delivery. The following sections include some consideration of the limitations commonly encountered for typical radiation oncology software platforms and may act as guidelines in establishing a clinic's individual summary of limitations.

###### B.6.1 Number of characters allowed

Software platforms assign a variable number of character maximums for objects. For example, Varian's Eclipse Treatment Planning System may assign a maximum of 16 characters for some objects (such as course names) but only 13 characters for others (such as treatment plan names). While the treatment planning system may allow a certain number of characters, it does not necessarily mean that the linear accelerator control console may allow the same maximum.^29^, ^30^

###### B.6.2 Forbidden characters

There are certain characters which are known to cause issues with particular computerized functions in radiation oncology applications. The forbidden characters may be unique to different software platforms. For example, the DICOM standard reserves the use of certain characters for special functions. These include the backslash “\”, the equal sign “=”, and the caret “^”.^31^, ^32^, ^33^ These are limitations of the DICOM standard, and none of these characters should be used in manual naming. A compilation of forbidden characters is found in Table 5.

**Table 5:** Notable characters recommended to not be used in the naming of treatment plan parameters

*Character Name*	*Symbol*
Backslash	\
Equal	=
Caret	^
Period	.
Exclamation Mark	!
At Sign	@
Pound	#

##### B.7 Image set naming

It is noted in these guidelines that image set naming is considered to be peripheral to the focus of this effort as long as the following information is readily available and linked to image sets:

- Modality (e.g., CT, PET, MRI, US)
- Submodality (e.g., T1 sequence MRI, T2)
- Origin (e.g., simulation CT, diagnostic CT, PET CT)
- Date of acquisition
- Any respiratory motion management details:
  ○ Retrospective gating studies should have clearly defined respiratory cycle assignments (for example, “CT_0_In” could represent the 0% inspiration bin).
  ○ MIPs, MinIPS, average, and other reconstruction techniques should be defined.

Furthermore, should the nature of the imaging be such that it directly impacts the radiotherapy delivery, then this should be made apparent in the information linked to the image sets. As an example, consider the scenario in which a patient requires re‐CT simulation during the course of delivery with an associated treatment plan revised to this new CT simulation. For such a situation, the date and intent of this CT should be made explicit.

##### B.8 Quick reference guide

A quick reference summary is provided in Table 4.

## IV. DISCUSSION

**Figure 4:**
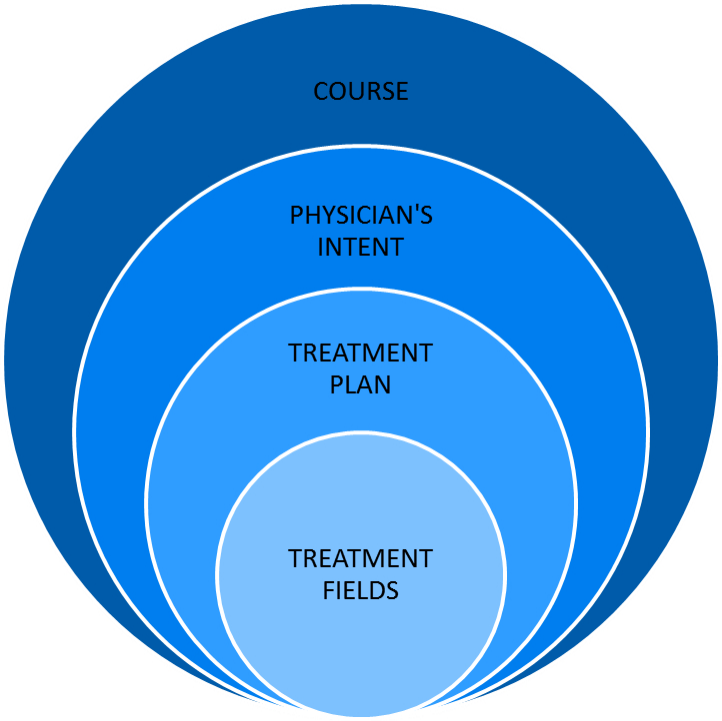
Organizational layout of the general guidelines for standard treatment naming for a radiation oncology clinic. The general guidelines for standard treatment naming for a radiation oncology clinic adheres to an organizational structure which mimics the treatment planning workflow. A short preamble is first presented (not illustrated here) describing the appropriate format for the naming of anatomical structures (described first because this format will propagate throughout the following sections). Next is described the format for naming of the treatment course. This is followed by the physician's intent (or prescription) to which a treatment plan will be generated which will utilize treatment fields. Thus, each subsequent step may be considered to be a subset of the previous step.

The guidelines presented here are organized such that the sections at the beginning discuss broader aspects of radiotherapy treatment planning. As the document progresses, the sections become more focused on the detailed aspects of the treatment planning process. The intent behind this organization is to mimic the natural workflow of the treatment process. To illustrate, note that the treatment course is considered first followed by the physician's intent after which a treatment plan is generated containing treatment fields. Therefore, each subsequent step may be considered a subset of the previous. Figure 4 illustrates this organizational layout.

Several studies in the literature have addressed naming standardization of individual classes of items specific to radiation oncology, such as targets and organs at risk naming^34^, ^35^, ^36^, ^37^ as well as standardization of tumor nomenclature.^(38)^ In a collaborative study of several radiation oncology groups, Santanam et al.^(34)^ reported the standardized naming conventions in radiation oncology. The guidelines included nomenclature for target volumes (clinical target volume, internal target volume, planning target volume), organs at risk, and planning organ‐at‐risk volumes. It also offered rules for specifying laterality and margins for different structures. The authors suggested that standardized naming guidelines are integral to compare dosimetry among datasets and promote international agreement in all aspects of radiation oncology.^(34)^ Less attention in the literature has focused on the objectives of the present study, specifically, standardizing methodology and strategy in radiation oncology with a particular emphasis on course, plan, and field naming.

Multiple approaches may be formalized regarding the standardization of the naming of anatomical structures and sites related to the treatment of radiotherapy patients. The goal of our guidelines was to consider the pros and cons of these unique approaches and specify a single approach to be universally implemented by the department. Adoption of the consensus developed by this methodology facilitated the creation of standardized templates based on disease site.

One example is for trigeminal neuralgia, which is a condition with uniform presentation and radiation treatment plans. Within the planning system, there now is a “SRS_Rt_Trig_Nerve” and a “SRS_Lt_Trig_Nerve” template. Choosing either one now prepopulates the site, laterality, course, plan, energy, treatment technique, and field definition. These fields follow the agreed upon capitalization, abbreviation, and nomenclature standards. Standard structures populate including: Rt_Trig_Nerve, PTV_Rt_Trig_Nerve, Brainstem, Rt_Cochlea, Lt_Cochlea, Brain, Body, Rt_optic_nerve, Lt_optic_nerve, Chiasm, Rt_eye, and Lt_eye. Additionally, these structures have predefined DVH limits for planning.

Anatomical naming may be used in the naming of parameter types such as the physician's intent, treatment course, organs at risk, and treatment plan and related fields. The same formalism should be adopted for all components where an anatomical specification is appropriate. It is noted that different parameters of the treatment planning workflow may require additional characters in the form of prefixes, suffixes, and additional laterality specifications. For example, consider a treatment planning system that is limited by a maximum number of characters of 13. A contoured mediastinum structure can be entitled fully as, for example, “Mediastinum”. Additional prefixes would exceed the maximum 13 allowable character spaces. Thus, one may consider the abbreviation of this course name to “C1_Mediastinm” or termination to “C1_Mediastinu” or any other variation that meets the limitation of the treatment planning system for this parameter type.

Single letter specifications may not always be advisable as redundancies of certain letters may cause confusion. For example, “left” and “lateral” would both require the same single letter specification. This necessitates the use of “Lat” for lateral reserving “L” for left. Furthermore, capitalizations are a necessity for laterality specifications as certain lower‐case letters may resemble upper case letters for certain fonts that may be employed in the radiation oncology clinic. For example, “I” may appear to be a lower‐case “ell” which could be interpreted to indicate a left laterality specification, or it could be perceived as an upper‐case “eye” indicating an inferior laterality specification, or it could be read as the numerical value of “one”. In typography, this is referred to as a homoglyph in which two characters cannot be differentiated by quick visual inspection (another example are the characters zero and the letter O for certain fonts and handwriting).^(39)^

This is taken a step further by suggesting that team members make an extra effort to utilize consistent units to further reduce the risk of miscommunication. Klein et al.^(18)^ cites several clinical examples of situations wherein the confusion from the use of units or unit signs led to mistreatments. If a patient alignment shift is verbally communicated to a colleague performing that shift on the patient setup, and it happens to be read to them as “2”, then it will make an order of magnitude difference whether or not that is a 2 millimeter or centimeter shift or a positive or negative “2”. In another situation, a physicist may read off a field size of “20” (intending 20 mm) in a radiosurgery treatment situation, and a therapist may set a field size of 20 cm that would ultimately result in a gross mistreatment.

In order to achieve a successful implementation of these naming conventions, it was recognized that the composition committee should consist of a finite number of contributors representing the multiple disciplines and that a much larger team of radiation oncology team members would need to buy‐in to this process. The high participation rates of the surveys associated with this effort indicate that involvement in the decision‐making process was able to be extended beyond the composition committee. By using a feedback‐based, iterative survey approach to widen participation, it was found that the nature of the guidelines disseminated to the clinical department were not unexpected. Rather, the team members had already been an integral part of the process at developing the final product and, thus, we expected to find greater implementation success as compared to procedural change that might come about without this extra buy‐in mechanism. Opening the development phase to a wider scope of participants permitted additional buy‐in, while the focus of the decision‐making committee allowed the study to reach a conclusion in a realistically finite period of time.

**Figure 5:**
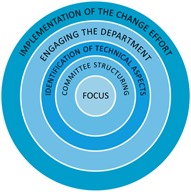
The modified buy‐in layered model. This illustration represents an approach to successfully implementing a change with a radiation oncology clinical department. The focus of this effort was the composition of a set of guidelines detailing the standardization of naming convention within the radiotherapy practice. This composition was carried out by carefully selecting the composition and decision‐making body comprised of representation of all of the end‐users of the product. This body was responsible for identifying and defining the limitations due to technical aspects involved with naming in a realistic clinical environment. Wide‐scope buy‐in was achieved through engaging a wider audience via the use of an iterative survey‐based approach. These surveys served multiple purposes including accurately identifying naming preferences, placing those preferences within the technical limitation framework, and garnering participation in an ultimate change in practice. This method was sensitive to the realistic emotional and situational hurdles involved in implementing such a change. The outer‐most layer represents the conclusion of this study in the clinical implementation of the naming conventions.

This methodology may best be described as modified from the so‐called buy‐in layered model as described by Campbell.^(40)^ Figure 5 depicts a set of guidelines for standardizing naming conventions in a radiation oncology clinic. The center represents the end‐product. The next layer is an identification of the individuals and/or groups that would be affected by a change, including physicians, therapists, physicists, dosimetrists, and nurses. This list also incorporates professionals such as administrators, billing specialists, and lawyers. The next layer is an identification of the technical aspects involved in radiation oncology. The next successive layer is engaging the department as achieved by the survey participation. The outermost layer is the implementation of the change effort. Successful communication facilitates the interaction of each layer.

The focus of the guidelines presented here has been on the naming of major items of the treatment plan that are commonly referenced cross‐disciplinary and used throughout the treatment workflow. The naming of treatment fields is used as a safety feature in the delivery of radiation by many different members of the radiation oncology team with varied backgrounds in training and experience and must, therefore, be able to provide meaningful and verifiable information subjected to multiple levels of checks performed prior to the delivery of any radiation.

## V. CONCLUSIONS

A standard nomenclature for treatment plan parameters in a radiation oncology environment is necessary to help mitigate the risk of patient mistreatment resulting from confusion in the interpretation of plan details. This study incorporates a multidisciplinary perspective to establish methodologies for standard treatment naming. The guidelines are realistically implementable for a typical radiation oncology environment. A future goal will be to evaluate the efficacy of the guidelines presented in this study in providing a naming standard that is readily adopted in a radiation oncology institution.

## ACKNOWLEDGMENTS

We acknowledge Norton Healthcare for their continued support, as well as the Associates in Medical Physics, LLC.

## COPYRIGHT

This work is licensed under a Creative Commons Attribution 4.0 International License.

## Supporting information

Supplementary Material Files

Supplementary Material Files
