# Supplementary material for: Guidelines for treatment naming in radiation oncology
Source: J Appl Clin Med Phys. 2015 Nov 7;17(2):123–38. doi: 10.1120/jacmp.v17i2.5953 (PMC5874902; doi:10.1120/jacmp.v17i2.5953)
Supplement: Supplementary file 1 — Supplementary Material Files [file ACM2-17-123-s001.doc]

**Guidelines for Treatment Naming in Radiation Oncology**

Travis R. Denton, D.M.P.,^1,2*^, Lisa B.E. Shields, M.D.^1,3,4^ Michael Hahl, M.D.^1^ Casey Maudlin, B.S.^1^

Mark Bassett, Ph.D.,^1^ Aaron C. Spalding, M.D., Ph.D.^1,4^

*^1^ The Norton Cancer Institute Radiation Center, Norton Healthcare, Louisville, KY*

*^2^ Associates in Medical Physics, LLC, Greenbelt, MD*

*^3^ Norton Neuroscience Institute, Norton Healthcare, Louisville, KY*

*^4^ The Brain Tumor Center, Norton Healthcare, Louisville, KY*

* To whom correspondence should be addressed:

Travis R. Denton, D.M.P, D.A.B.R.

The Norton Cancer Institute Radiation Center

Norton Healthcare

676 S. Floyd St., Suite 130

Louisville, KY 40202

Phone: (502) 629-4555

Fax: (502) 629-4599

E-mail: travis.r.denton@gmail.com

**Running Head:** Guidelines for Treatment Naming
