# Supplementary material for: Guidelines for treatment naming in radiation oncology
Source: J Appl Clin Med Phys. 2015 Nov 7;17(2):123–38. doi: 10.1120/jacmp.v17i2.5953 (PMC5874902; doi:10.1120/jacmp.v17i2.5953)
Supplement: Supplementary file 2 — Supplementary Material Files [file ACM2-17-123-s002.doc]

**Guidelines for Treatment Naming in Radiation Oncology**

**Running Head:** Guidelines for Treatment Naming

**Guidelines for Treatment Naming in Radiation Oncology**

**ABSTRACT:** Safety concerns may arise from a lack of standardization and ambiguity during the treatment planning and delivery process in radiation therapy. A standard nomenclature in radiation oncology is important for mitigating the risk of patient mistreatment due to confusion in the interpretation of plan details. The continuous quality improvement (CQI) committee of the department of radiation oncology meets monthly to monitor opportunities to improve patient safety. Review of a patient safety reporting system (PSRS) submissions as part of CQI revealed recurring reports related to confusing or ambiguously named planning parameters. A naming standard could help mitigate the risk of patient mistreatment that may arise from unclear naming practices. A multi-discipline, multi-clinical site consortium was established to create a guideline for standard naming. Guidelines for standard treatment naming were developed. The requirements imposed on the guidelines included that the standards be: realistically achieved, concise, straightforward, practical, definitive, adaptable, relevant, flexible, and assessable given the limitations from a resource, technical, and compliance perspective. General guidelines for standard treatment naming in radiation oncology are presented. This multidisciplinary study provides a clear, straightforward, and easily implemented protocol for the radiotherapy treatment process. Standard nomenclature facilitates the safe means of communication between team members and complements the CQI philosophy in radiation oncology.

**KEY WORDS:** Radiation Therapy, Naming Convention, Nomenclature, Record and Verify, Quality Management, Quality Improvement, Standardizing

**I. INTRODUCTION**

Radiation oncology relies on the principles of process improvement as a means of promoting quality and development in the delivery of the therapies involved in clinical practice. Standardization of workflow, processes, and personnel training requires integration of multiple technical and human components. One way to ensure uniform and safe treatment deliveries compliant with the radiation oncologists’ intents for all of these levels of the treatment care path is to establish the use of consistent and meaningful nomenclature.^1-3^ Nomenclature, as defined for the radiation oncology environment, is the devising of terms for components of the radiation prescribing, simulation, planning, or treatment process. These terms should have a basis in logic and be reproducible outside of an individual or local group within the clinic.^4^ Standards used in healthcare have been found to significantly improve the accuracy of achieving the physician’s intent of treatment, the effectiveness of communication between individual radiation oncology team members and software-based platforms, and the quality of patient record keeping.^5-12^

Inconsistencies or poorly chosen naming practices in a radiation oncology clinic may lead to confusion in the interpretation of important details which may cause patient treatment errors or the misadministration of therapy.^13-17^ It has been reported in the literature that lack of naming standardization contributes to the incident of event reporting via mechanisms such as PSRS.^18^ Changes in practice must be based on a careful assessment of the needs of that change as well as understanding of the causes that could lead to treatment errors.^19^ The PSRS in conjunction with CQI programs provide an excellent mechanism from which to evaluate, present, and drive valid changes in practice centered around improving the safety and efficacy of patient treatment.^20-22^ In general, naming conventions can help cope with the ever expanding levels of sophistication of modern treatment simulation, planning, and delivery systems and increase the safety culture of a clinic.^7,22-24^

There can be difficulties in universal adoption of naming conventions.^25^ The successful implementation of a convention requiring a change in practice in a clinic must overcome cognitive, attitude, professional, practicality, and lack of organization barriers.^19,26^ The challenge is illustrated by noting the gap that may exist between research recommendations, clinical practice guidelines, and actual clinical practice in a clinic setting.^19^

We conducted this study to identify uniform naming conventions to reduce errors in a radiation department treating at multiple locations with external beam, brachytherapy, and unsealed sources. We present here our quantitative, iterative methodology for improved patient safety based on input from clinical and system quality staff.

**II. MATERIALS AND METHODS**

The department in this study operates five linear accelerators and provides services for external beam radiotherapy, intensity modulated radiotherapy (IMRT), stereotactic radiosurgery, stereotactic body radiotherapy, high dose rate brachytherapy, low dose rate brachytherapy, Y-90 radiopharmaceutical therapy, and I-131 radiopharmaceutical therapy for three separate physical clinic sites operating under the umbrella of a single institution. The patient management system used intra-departmentally is Aria (Varian Medical Systems, Palo Alto, CA), while the inter-hospital department electronic medical record system is EPIC (Epic Systems Corporation, Madison, WI). The treatment planning systems utilized are the following: Eclipse (Varian Medical Systems, Palo Alto, CA), iPlan (Brainlab AG, Feldkirchen, Germany), BrachyVision (Varian Medical Systems, Palo Alto, CA), and VariSeed (Varian Medical Systems, Palo Alto, CA).

The institution’s CQI committee recognized over a six month period that a number of reports filed to the hospital’s PSRS were related to incorrect, ambiguous, or uncertain naming events for plan parameters specific to patients’ treatments. The committee determined that an institution-wide, multi-disciplinary derived naming standard would help mitigate the potential risk for mistreatment that may result from ambiguously naming treatment parameters. The committee was faced with numerous challenges including: modifying or discontinuing the use of pre-established yet incomplete or non-universal naming practices adopted individualistically, un-ingraining habit-based naming practices not in keeping with the philosophy of safety and universality that the standardization protocol would embody, and promoting input from all team members that would be responsible for the successful and complete adoption of the standardization guidelines.

The committee commissioned an effort to provide a naming convention that could be implemented by all of the radiotherapy staff members. The group designated to compose the general guidelines in standard treatment naming presented in this study was formed on January 8, 2014. The report was implemented by the institution on February 1, 2015. The entire scope of the CQI is described in Table 1, while this manuscript focuses on phase I of this table. The committee consisted of three committee members (Physicist/Dosimetrist, Radiation Therapy Technologist, and Radiation Oncologist) and one team leader (Director of Radiation Oncology Services). We gathered input using electronic and anonymous surveys disseminated on a regular basis with each survey addressing a different sub-component of the scope of the project. The purpose was to encourage maximum participation by minimizing the time needed to complete each survey and to provide clear start and end dates for the timely completion of each survey.

Surveys were disseminated regarding three areas of interest: course naming, treatment plan naming, and treatment field naming. Each survey was available for one week and was followed immediately by the next survey. Designed to be able to be completed in approximately five minutes, each survey consisted of three to five questions consisting of multiple choice responses, free-form comment fields, and ranking question types.

A scoring system was used in which options receiving greater preference status in the responses were assigned greater scores. The scores were summed for each option and divided by the sum of the number of responses for that question. For each, if an option (considered against three other options) received one most preferred ranking, two second most preferred rankings, zero third preferred rankings, and three least preferred rankings for a question with six responses gathered, then the score would be:

*.-*
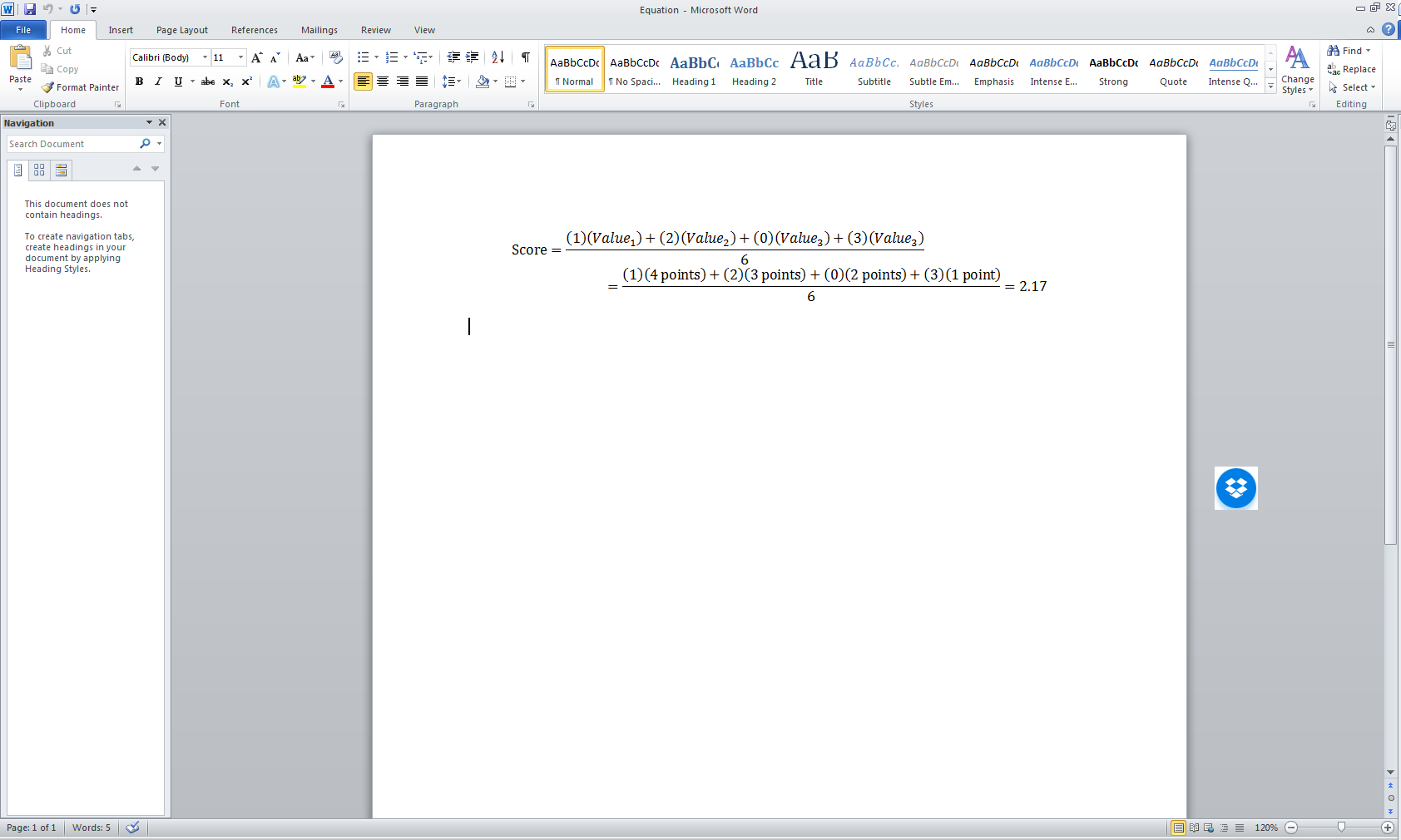


It was the intent of the committee to be able to take advantage of the expertise and experience of this extended group of professionals and address previously unconsidered issues that were pointed out during the course of collecting these surveys. The responses by the users were recorded anonymously, and the compiled results from the survey were not shared outside of the standardization committee prior to the release of the general guidelines for standard treatment naming for the radiation oncology department.

A system was created for naming standardization of the following: anatomical sites, treatment course, treatment prescription, treatment plan, and treatment field. Additional concepts were addressed including, the use of capitalization, specification of laterality, course naming in the event of multiple sites being treated within the same course of treatment, primary versus boost planning, the use of bolus, revisions for plans, image-guidance field naming, forbidden characters, and standard units for commonly used physical quantities in radiation oncology practice. This formalization was made with the understanding of the limitations derived during the use of the patient management software employed in a typical radiation therapy environment and with the interests of risk mitigation at the forefront of the establishment of the nomenclature as used by all members of the radiation oncology team.

The report had to present a naming standard that was: realistically implementable, sufficiently brief, readable and functional,^27^ inclusive of concrete and specific statements,^28^ adaptable to current practices, relevant to current practices, representative and embodying quality communication, mindful of resource implications,^29^ clinically flexible,^4^ and evaluable via retrospective audit post implementation.^30^ The flexibility requirement, mentioned above, is illustrated via the example that structure names for two hypothetical structures: “limb” and “extremity” could be meaningfully interpreted by a colleague to mean the same thing without causing confusion. However, the use of structure names such as “arm” and “limb” could cause confusion. Thus, the convention should allow for a reasonable degree of flexibility but not to the extent that it allows for naming confusion. This requirement for flexibility was integral in assuring the successful implementation of the convention as had been cited in other examples of efforts to implement changes in practice in a clinic setting.^4^ These requirements promoted confidence in adopting of the end result.^31^

**III. RESULTS**

**A. Survey Participation**

The committee solicited input from all key stakeholders with brief electronic surveys, with a completion rate of 73.0%. These surveys and high completion rate allowed the iterative development of uniform naming standards, based on using more general terms for courses and more details for treatment plan parameters (Figure 1). The surveys were designed with a limited number of choices for two reasons. First, this allowed quantitation of the results. Second, it facilitated adoption of the recommended choice. Figure 2 demonstrates the weighted responses from the survey used to obtain a consensus for naming a course of treatment. This clearly shows the department preference for including both the number, laterality, and site within a course designation. Figure 3 outlines the results with a larger survey demonstrating identification of the most critical elements of the fields used in a treatment plan to include number and orientation.

**B. General Guidelines for Standard Treatment Naming for a Radiation Oncology Department**

**B.1 Naming of Anatomical Sites**

The formalism proposed here for the naming of anatomical sites may be extended into all of the radiotherapy clinical components related to treatment planning and delivery where an anatomical specification is appropriate and should remain consistent throughout the documentation for a radiation oncology treatment plan. A summary of these conventions is provided in Table 2.

**B.1.1 Regarding the Use of Capitalization**

In writing compound words in the treatment planning and delivery workflow, it is recommended that the user use capitalized words for the start of each word with no spacing between words. This can extend to abbreviations with the first letter in the abbreviation being capitalized and the following in lower-case. For example, should the user wish to abbreviate the word “right” to a two letter abbreviation, the user would write: “Rt”.

**B.1.2.1 Writing Laterality**

It is recommended that, if only one laterality is needed to be specified, that the longer form of the abbreviation be used (for example, “RtLung” instead of “RLung”). But, if multiple lateralities need specification, then the shorter is allowable (e.g. “RPO”). Also, if it is possible to include the entire name, then it is acceptable to do so with the understanding that other steps of the treatment workflow may require abbreviation.

**B.1.2.2 Laterality with Anatomical Site**

When writing a laterality combined with an anatomical site, the laterality shall be used as a prefix to the structure name. For example, in consideration of a right lung, the naming shall be “RtLung” (and not, “LungRt”).

**B.1.3 Summary of Anatomical Site Guidelines**

An anatomical site:

- Should use capitalization for the start of each new word (including laterality specifications).
- Should be used consistently throughout the plan records (though abbreviations are acceptable when necessary).
- Should list laterality specifications written before the anatomical site.

**B.2 Course Naming**

Course naming is recommended to include both a course designation character in addition to a course number.

**B.2.1 Iterative Course Naming**

Patients may require multiple courses of treatment for typical radiation oncology clinics. For the purposes of these guidelines with regards to radiation oncology, a treatment course is defined to:

- Be a fixed number of regular radiation derived medical treatments.
- Be predetermined and explicitly specified (in terms of both number of fractions, dose per fraction, and frequency for fractions) by the physician prior to the delivery of radiation treatment.
- Have a clearly defined intended target(s) of treatment assigned to specific treatment plan(s).
- Include only the treatment plans that are predetermined to be delivered at the initiation of the treatment regime developed by the physician (including boost plans).
- Include multiple plans for a patient if those plans are intended to be delivered concurrently or in a consecutive scheduled succession.
- Include multiple plans for a patient if those plans share a common CT simulation acquired prior to treatment delivery.
- Include multiple plans for a patient if those plans constitute, as a whole, the intended care for a particular patient.

As such, the following requirements are recommended upon the naming of courses:

- The course names shall have a clear chronological order.
- The courses should rely on automated naming as much as possible to minimize the need for treatment planning system user intervention and to reduce the potential errors that could arise from having to modify course names.
- The course name should include a brief descriptor of the treatment objective (whether it be defining the explicit treatment site or relating some manner of the treatment technique in a clear and meaningful way).

In light of this definition and these requirements, course naming, for these guidelines, recommends the standardization via use of the “C#” formalism with the addition of the anatomical site to be treated in the relevant course of treatment appended in the name. For example, for a hypothetical patient’s second course of treatment for a left lung site, the course name would be: “C2_LtLung”.

Iteration of the course number is recommended to be continuous for all treatment sites and with the numbering not re-started if a new treatment site were to require treatment. Thus, if this hypothetical patient’s first course of treatment had been to the right breast, then their first course would be entitled: “C1_RtBreast”.

**B.2.2 Course Naming in the Event of Multiple Sites within the Same Course**

If multiple treatment plans are necessary for a patient’s treatment, as pre-determined by the physician prior to the onset of radiation therapy, these plans should be included in the same course of treatment. The following two sections describe how to address the naming of a course that includes multiple plans of treatment meeting the above criteria.

**B.2.2.1 Sites are in a Shared Anatomical Site**

If multiple plans are necessary within the same course of treatment and are to be delivered within a shared anatomical site, then the shared anatomical site should be included in the course naming. For example, for a patient receiving two treatment plans to a left frontal and right parietal metastatic lesion, the course name should be written as: “C1_Brain” or some variation of this indicating that the course of treatment contains cranial treatment plans. As another example, consider a right breast treatment which contains both a primary and a boost treatment. The course name for this patient should be written as: “C1_RtBreast”.

**B.2.2.2 Sites are not in a Shared Anatomical Site**

For treatment plans within the same course and not delivered to a common site, the treatment course name is recommended to include a reflection of the general intention to treat multiple sites with the specificity of the site reserved for the physician’s intent and the treatment plans. For example, a patient receiving a treatment to metastatic bone sites in both their pelvis and left humerus should have a course written as: “C1_MultipleMets” with plan names of: “Pelvis” and “LtHumerus”.

**B.3 Naming for Treatment Prescriptions**

The requirements for physician’s intent naming as recommended by these guidelines include the following:

- The anatomical site must be clearly stated.
- The name shall adhere to the specifications detailed in section III.B.1.
- Laterality (where appropriate) shall be clearly stated and adhere to the format recommended in section III.B.1.

**B.4 Treatment Plan Naming**

The requirements for treatment plan naming are as follows:

- The plan name should match the physician’s intent exactly.
- If possible, a qualifier should be affixed to the plan name to indicate technique.

For example, if two plans are generated for a right lung treatment site with the first attempting IMRT as a treatment technique and the other volumetric modulated arc therapy (VMAT), then the two plans could be written as: “RtLung_IMRT” and “RtLung_VMAT”, respectively.

**B.4.1 Primary versus Boost Plan Naming**

When naming boost plans, it is the recommendation of these guidelines that a suffix of “_bst” be added to the standard format for plan naming to indicate that this plan corresponds with the physician’s intent for a boost plan. When naming primary plans in the same course, no additional suffix is necessary. Simply follow the guidelines in the preceding section. For example, for a patient receiving both a primary and a boost treatment plan for a right breast treatment, the plan names could be written as: “RtBreast” and “RtBreast_bst”, respectively.

**B.4.2 Bolus in Plan Naming**

For treatment plans utilizing bolus, the use of bolus should not be specified in the treatment plan name as recommended here in the interest of character limitations, possible ambiguity, and the risk for mistreatment if the user should fail to adhere to the naming convention. Rather, the use of bolus should be explicitly included in the physician’s intent, setup notes, and any interlock or sign-off fields at the treatment delivery workstation.

**B.4.3 Plan Revisions**

When revisions to treatment plans are deemed necessary after the initiation of the treatment course, the default naming convention used in the treatment planning system is recommended to be sufficient. For example, in Varian’s Aria environment, the original plan’s name is reproduced but also appended with “:#” with the numerical value increasing with each additional plan revision (to “:2”, “:3”, etc.). Relying on this automation will reduce errors due to transcription mistakes and will help preserve the continuity of treatment in a sufficiently clear manner for the purposes of treatment audit. Furthermore, the original plan name will remain available to be matched against the physician’s intent.

**B.4.4 Multi-Stage Plan Naming**

For plans that require multiple stages of delivery, the plan name for each stage is recommended to have a qualifier indicating that intent. For example, for a treatment followed by a reduction in treatment volume, a subsequent plan could be appended with “_Reduce1” to indicate the first reduction needed and so forth.

**B.4.5 Merged Fields**

For certain linear accelerators and the designs of their treatment heads, it may become necessary to merge treatment fields with common gantry/couch/collimator parameters but needing a shift in the multileaf collimators to allow for a different collimation of the treatment field. For such merged treatment fields, the post-merged treatment field is recommended to be appended with the suffix of “FinF” (field-in-field). For example, merging treatment fields for a left breast plan would result in a field name of “LtBreastFinF”.

**B.5 Treatment Field Naming**

Treatment field naming may be written with patient-specific anatomical orientation and iterative indicators to relate efficient field ordering and provide additional differentiation between field names. To further promote delivery efficiency and patient throughput, the field numbering prefix can begin with “01” and increase iteratively throughout the available fields in the treatment plan. Furthermore, this numbering can continue with additional plans delivered in the treatment course, and with additional plans required of future courses of radiotherapy.

**B.5.1 Dynamic Gantry Treatment Fields**

For arc-based treatment fields, it is the recommendation of these guidelines that a hyphen be used to denote gantry motion. Thus, if, during the delivery of the treatment field, the gantry were to rotate from a left-posterior-oblique (LPO) to a right-anterior-oblique (RAO) patient-specific orientation, then the field can be entitled: “01_LPO-RAO” with the hyphen indicating the transition of the field from its starting to the field conclusion position. It is furthermore proposed that the hyphen *only* be used to indicate a transition or a range. In all other cases, the underscore (“_”) symbol should be used.

**B.5.2 Image-Based Setup Field Naming**

Image alignment field naming can be also be defined using these guidelines. For such fields, it is recommended that both the imaging modality and the patient-specific orientation to which the digitally reconstructed radiograph (if applicable) was derived be explicitly stated in the name. For example, various kilovoltage (kV) setup fields and a cone-beam CT (CBCT) can be routinely named as follows: AP_kV, Rt_kV, Lt_kV, and CBCT.

**B.6 Allowed and Forbidden Characters Typically Encountered in Patient Management Systems**

Computerized patient management and treatment planning systems inherently include limitations on the number and types of characters available. Importantly, the limitations are not uniform across software platforms. However, information embedded in the naming of different plan parameters needs to be able to cross multiple platforms along the process from simulation to treatment delivery. The following sections include some consideration of the limitations commonly encountered for typical radiation oncology software platforms and may act as guidelines in establishing a clinic’s individual summary of limitations.

**B.6.1 Number of Characters Allowed**

Software platforms assign a variable number of character maximums for objects. For example, Varian’s Eclipse Treatment Planning System may assign a maximum of 16 characters for some objects (such as course names) but only 13 characters for others (such as treatment plan names). While the treatment planning system may allow a certain number of characters, it does not necessarily mean that the linear accelerator control console may allow the same maximum.^32,33^

**B.6.2 Forbidden Characters**

There are certain characters which are known to cause issues with particular computerized functions in radiation oncology applications. The forbidden characters may be unique to different software platforms. For example, the DICOM standard reserves the use of certain characters for special functions. These include the backslash “\”, the equal sign “=”, and the caret “^”.^34-36^ These are limitations of the DICOM standard, and none of these characters should be used in manual naming. A compilation of forbidden characters is found in Table 3.

**B.7 Image Set Naming**

It is noted in these guidelines that image set naming is considered to be peripheral to the focus of this effort as long as the following information is readily available and linked to image sets:

- Modality (CT, PET, MRI, US, etc.)
- Submodality (e.g. T1 sequence MRI, T2, etc.)
- Origin (simulation CT, diagnostic CT, PET CT, etc.)
- Date of Acquisition
- Any respiratory motion management details:
  - Retrospective gating studies should have clearly defined respiratory cycle assignments (for example, “CT_0_In” could represent the 0% inspiration bin).
  - MIPs, MinIPS, average, and other reconstruction techniques should be defined.

**B.8 Quick Reference Guide**

A quick reference summary is provided in Table 2.

**IV. DISCUSSION**

The guidelines presented here are organized such that the sections at the beginning discuss broader aspects of radiotherapy treatment planning. As the document progresses, the sections become more focused on the detailed aspects of the treatment planning process. The intent behind this organization is to mimic the natural workflow of the treatment process. To illustrate, note that the treatment course is considered first followed by the physician’s intent after which a treatment plan is generated containing treatment. Therefore, each subsequent step may be considered a subset of the previous. Figure 4 illustrates this organizational layout.

Multiple approaches can be formalized regarding the standardization of the naming of anatomical structures and sites related to the treatment of radiotherapy patients. The goal of our guidelines was to consider the pros and cons of these unique approaches and specify a single approach to be universally implemented by the department. Anatomical naming may be used in the naming of parameter types such as: the physician’s intent, treatment course, organs at risk, and treatment plan and related fields. The same formalism should be adopted for all components where an anatomical specification is appropriate. It is noted that different parameters of the treatment planning workflow may require additional characters in the form of prefixes, suffixes, and additional laterality specifications. For example, consider a treatment planning system that is limited by a maximum number of characters of thirteen. A contoured mediastinum structure can be entitled fully as, for example, “Mediastinum”. Additional prefixes would exceed the maximum 13 allowable character spaces. Thus, one may consider the abbreviation of this course name to “C1_Mediastinm” or termination to “C1_Mediastinu” or any other variation that meets the limitation of the treatment planning system for this parameter type.

Single letter specifications may not always be advisable as redundancies of certain letters may cause confusion. For example, “left” and “lateral” would both require the same single letter specification. This necessitates the use, as illustrated in table b, of “Lat” for lateral reserving “L” for left. Furthermore, capitalizations are a necessity for laterality specifications as certain lower-case letters may resemble upper case letters for certain fonts that may be employed in the radiation oncology clinic. For example, “I” may appear to be a lower-case “ell” which could be interpreted to indicate a left laterality specification or it could be perceived as an upper-case “eye” indicating an inferior laterality specification or it could be read as the numerical value of “one”. In typography, this is referred to as a homoglyph in which two characters cannot be differentiated by quick visual inspection (another example are the characters zero and the letter O for certain fonts and handwriting).^37^

This is taken a step further by suggesting that team members make an extra effort to utilize consistent units to further reduce the risk of miscommunication. Klein et al. cites several clinical examples of situations wherein the confusion from the use of units or unit signs led to mistreatments.^18^ If a patient alignment shift is verbally communicated to a colleague performing that shift on the patient setup, and it happens to be read to them as “2”, then it will make an order of magnitude difference whether or not that is a 2 millimeter or centimeter shift or a positive or negative “2”. In another situation, a physicist may read off a field size of “20” (intending 20 mm) in a radiosurgery treatment situation, and a therapist may set a field size of 20 cm that would ultimately result in a gross mistreatment.

In order to achieve a successful implementation of these naming conventions, it was recognized that the composition committee should consist of a finite number of contributors representing the multiple disciplines and that a much larger team of radiation oncology team members would need to buy-in to this process. The high participation rates of the surveys associated with this effort indicate that involvement in the decision making process was able to be extended beyond the composition committee. By using a feedback-based, iterative survey approach to widen participation, it was found that the nature of the guidelines disseminated to the clinical department were not unexpected. Rather, the team members had already been an integral part of the process at developing the final product, and, thus, we expected to find greater implementation success as compared to procedural change that might come about without this extra buy-in mechanism. Opening the development phase to a wider scope of participants permitted additional buy-in while confining the decision-making committee allowed the study to reach a conclusion in a realistically finite period of time.

This methodology may best be described as modified from the so-called buy-in layered model as described by Campbell.^38^ Figure 5 depicts a set of guidelines for standardizing naming conventions in a radiation oncology clinic. The center represents the end-product. The next layer is an identification of the individuals and/or groups that would be affected by a change, including physicians, therapists, physicists, dosimetrists, and nurses. This list also incorporates professionals such as administrators, billing specialists, and lawyers. The next layer is an identification of the technical aspects involved in radiation oncology. The next successive layer is engaging the department as achieved by the survey participation. The outermost layer is the implementation of the change effort. Successful communication facilitates the interaction of each layer.

The focus of the guidelines presented here has been on the naming of major items of the treatment plan that are commonly referenced cross-disciplinary and used throughout the treatment workflow. The naming of treatment fields is used as a safety feature in the delivery of radiation by many different members of the radiation oncology team with varied backgrounds in training and experience and must, therefore, be able provide meaningful and verifiable information subjected to multiple levels of checks performed prior to the delivery of any radiation

The PSRS used at the onset of this effort was insufficiently capable of reporting events from a radiation oncology perspective. This system utilized a framework of reporting that was derived from radiology practices. As such, it was found that the resulting reports provided similar organizational structuring to those needed by a typical radiation oncology clinic but ultimately lacked in the specificity needed to fully describe and effectively utilize the reports generated in the system. The PSRS presented herein is capable of catering to the language and reporting structure that may be useful for radiation oncology specific CQI efforts. A future goal associated with this study will be using this system to evaluate the efficacy of the guidelines presented here in providing an effective naming standard for this radiation oncology institution.

**V. CONCLUSION**

A standard nomenclature for treatment plan parameters in a radiation oncology environment is necessary to help mitigate the risk of patient mistreatment resulting from confusion in the interpretation of plan details. This study incorporates a multidisciplinary perspective to establish general guidelines for standard treatment naming. The guidelines are realistically implementable for a typical radiation oncology environment and represent the efforts of CQI.

**Acknowledgements:** We acknowledge Norton Healthcare for their continued support as well as the Associates in Medical Physics, LLC.

**Abbreviation List:** CQI: continuous quality improvement; PSRS: patient safety reporting system; IMRT: intensity modulated radiotherapy; VMAT: volumetric modulated radiotherapy; kV: kilovoltage; CBCT: cone-beam computed tomography.

**Figures:**

**Figure 1: The general-to-specific philosophy of plan parameter naming as applied to radiation oncology**

**
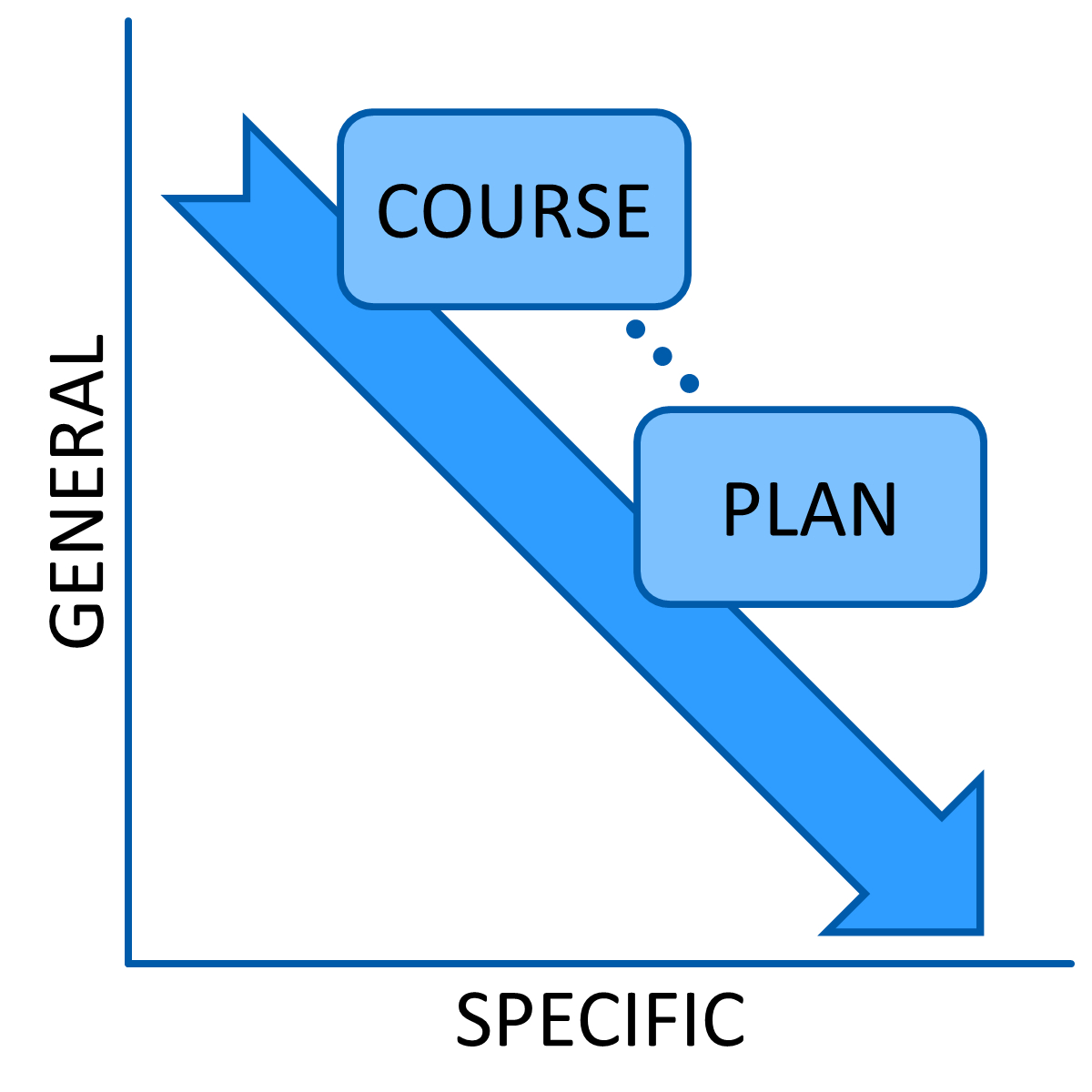
**

**Figure 2: Ranking preferences for four options of course naming standards**

**
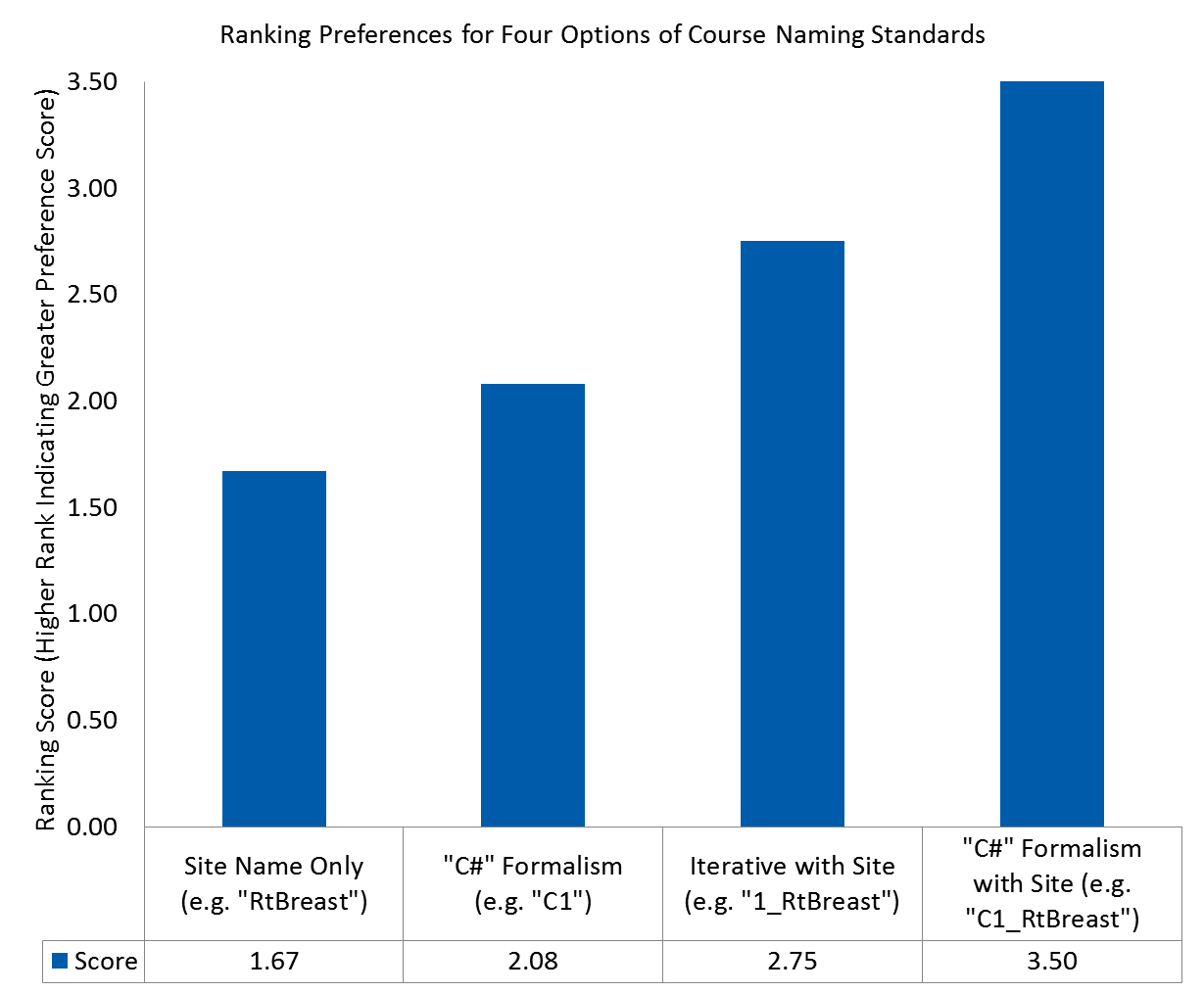
**

**Figure 3: Ranking preferences for six options of field naming standards**

**
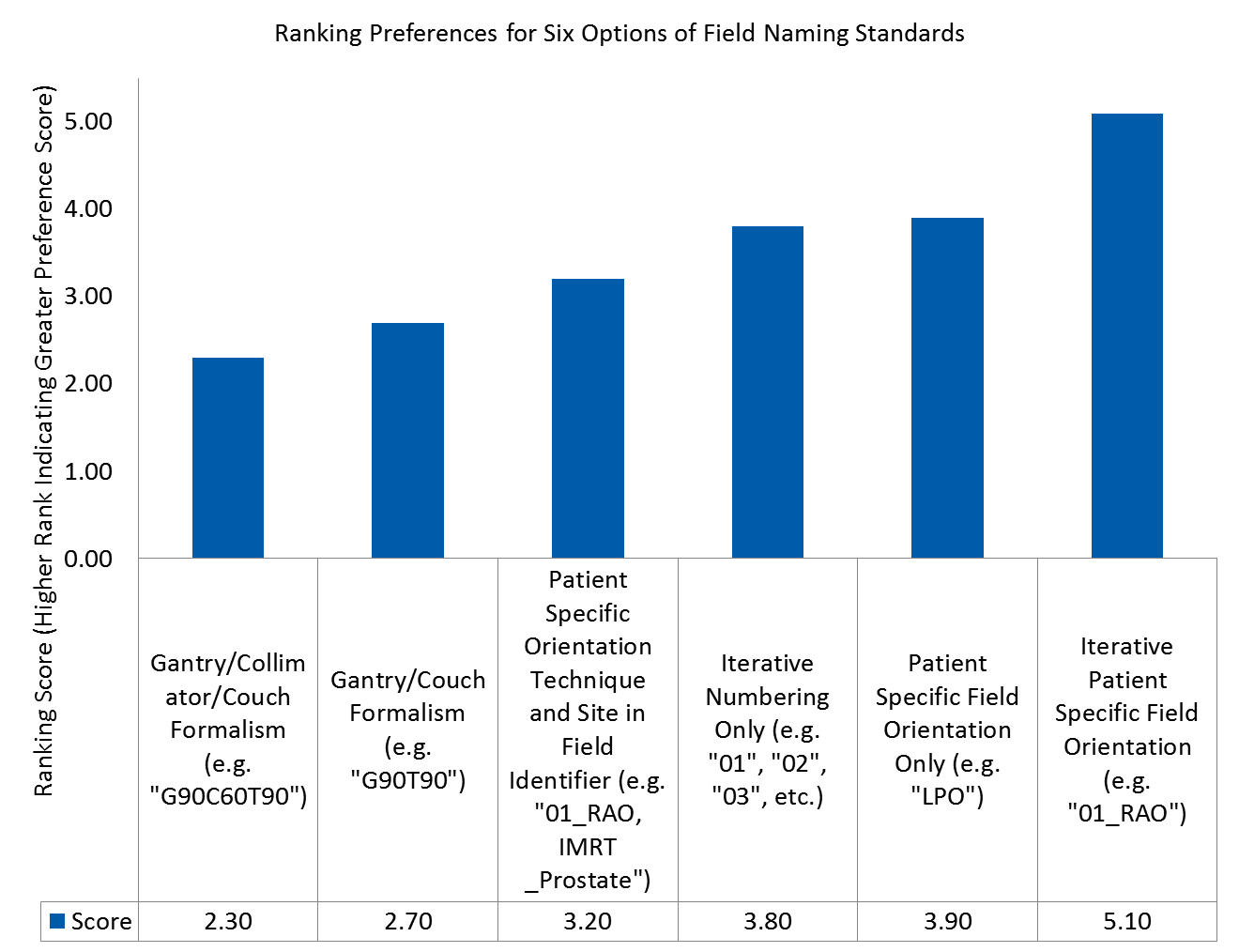
**

**Figure 4: Organizational layout of the general guidelines for standard treatment naming for a radiation oncology clinic**


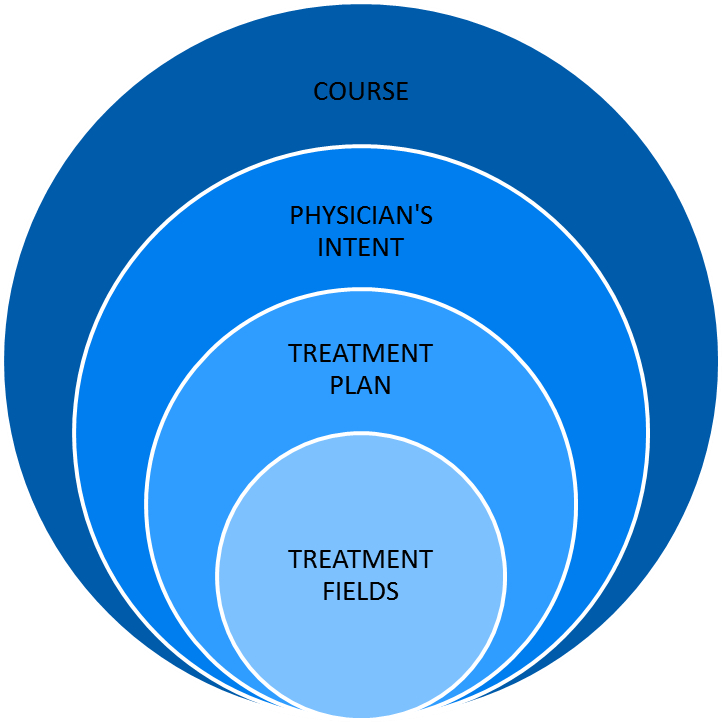


**Figure 5. The modified buy-in layered model**


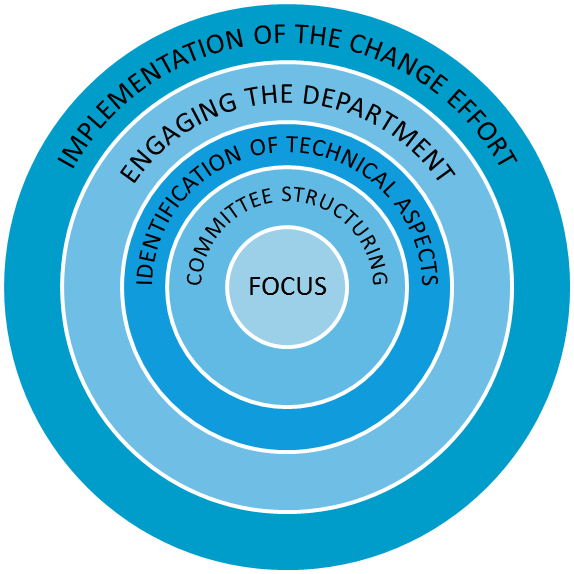


**Figure Legend:**

Fig. 1. The general-to-specific philosophy of plan parameter naming as applied to radiation oncology treatments

Course naming can be generalized to a greater degree to allow for accurate and meaningful naming while not demanding impossible naming restrictions. At the plan level though, a greater deal of specificity is required.

Fig. 2. Ranking preferences for four options of course naming standards

This plot shows the ranking, sorted in ascending order, for four options presented during the intra-departmental survey outreach. The greater scores are associated with the more preferred standards from the polling of the survey recipients within the department.

Fig. 3. Ranking preferences for six options of field naming standards

This plot shows the ranking, sorted in ascending order, for six options presented during the intra-departmental survey outreach specific to treatment field naming standards. The greater scores are associated with the more preferred standards from the polling of the survey recipients within the department.

Fig. 4. Organizational layout of the general guidelines for standard treatment naming for a radiation oncology clinic

The general guidelines for standard treatment naming for a radiation oncology clinic adheres to an organizational structure which mimics the treatment planning workflow. A short preamble is first presented (not illustrated here) describing the appropriate format for the naming of anatomical structures (described first because this format will propagate throughout the following sections). Next, is described the format for naming of the treatment course. This is followed by the physician’s intent (or prescription) to which a treatment plan will be generated which will utilize treatment fields. Thus, each subsequent step may be considered to be a subset of the previous step.

Figure 5. The modified buy-in layered model

This illustration represents an approach to successfully implementing a change with a radiation oncology clinical department. The focus of this effort was the composition of a set of guidelines detailing the standardization of naming convention within the radiotherapy practice. This composition was carried out by carefully selecting the composition and decision-making body comprised of representation of all of the end-users of the product. This body was responsible for identifying and defining the limitations due to technical aspects involved with naming in a realistic clinical environment. Wide-scope buy-in was achieved through engaging a wider audience via the use of an iterative survey-based approach which served multiple purposes including: accurately identifying naming preferences and placing those preferences within the technical limitation framework and for garnering participation in an ultimate change in practice being mindful of the realistic emotional and situational hurdles involved in implementing such a change. The outer-most layer represents the conclusion of this study in the clinical implementation of the naming conventions.

**Table Legend:**

Table 1. Global Scope of the Nomenclature Standardization Efforts

| RADIATION ONCOLOGY NOMENCLATURE SCOPE | | |
| --- | --- | --- |
| PHASE | PHASE TITLE | PHASE COMPONENTS/GOALS |
| I | Treatment Planning Parameters | Anatomical Site, Course, Treatment Plan, Treatment Fields, Standard Units of Physical Quantities, Forbidden Characters |
| II | Document Naming | Consultation, Consent, Orders, Visits, End-of-Treatment, Follow-Up |
| III | Treatment Activity Naming | Standardizing activity titles |
| IV | Billing | Providing clear billing guidelines |
| V | Treatment Care Path Template | For various treatment modalities (e.g. EBRT, IMRT, SBRT, HDR, etc.) |

Table 2. Summary of General Guidelines for Standard Treatment Naming for a Radiation Oncology Clinic

| NAMING LEVEL | NAMING RULES | EXAMPLES |
| --- | --- | --- |
| General Capitalization | Capitalize the first letter of each new word. | RightLung |
|  | Capitalize the first letter in each word of the abbreviated phrase. | RAO; LtObl |
| Laterality | Use only approved laterality abbreviations. |  |
|  | Specify laterality before structure name. | RtLung; LtOpticNerve |
| Course | Use the "C#" formalism. | C1, C2, C3, etc. |
|  | Specify the treatment objective following the "C#" formalism. | C2_LtLung |
|  | Include all plans within the same course number if plans are initially planned to be delivered concurrently or in physician directed succession. | C1_RightBreast; C1_Rbreast_bst |
|  | Include all plans within the same course number if plans treated share a planning CT set. |  |
|  | Include all plans within the same course number if the multiple plans constitute the intended treatment at the time of physician specification of the intents. |  |
|  | Begin a new course number if a new plan is unrelated to the original treatment or not part of the intended treatment regime of the original course |  |
|  | If multiple sites are being treated in the same course, list the common structure (if applicable). | C1_Brain |
|  | If multiple sites in the same course do not share a common structure, the treatment objective should be named to reflect the intention of treating multiple sites. | C3_MultipleMets |
| Prescribe Treatment | Anatomical site must be clearly labelled. |  |
|  | Laterality should be clearly labelled. |  |
| Treatment Plan | Plan name should consist of a component that matches the physician's intent exactly. |  |
|  | If possible, the plan name should include a qualifier suffix to indicate treatment technique. | RtLung_IMRT; RtLung_VMAT |
|  | For boost plans, a qualifier suffix should be added of: "_bst". | RtBreast_bst |
|  | No qualifier suffix is necessary for primary plans. |  |
|  | Plan revisions should follow automatic naming conventions if possible and should include annotation detailing the need for the revision. | Example: RtLung:2; RtLung:3; etc. |
|  | Multiple stage plans should include a suffix qualifier to the plan name indicating the specification of the stage. | Larynx_Quad1 |
|  | Field-in-field plan names shall include the suffix "FinF. | RtBreast_FinF |
| Treatment Fields | Iterating field numbers should be used. | 01, 02, 03, etc. |
|  | An anatomical-specific laterality indicator should be used. | 01_LPO |
|  | The hyphen symbol should be reserved for and only used for indicating transition. | 02_LPO-RAO (dynamic gantry treatment field example) |
| Setup Fields | The following field names can be used to denote setup fields: AP_kV, Rt_kV, Lt_kV, and CBCT. |  |

Table 3. Notable Characters Recommended to not be used in the Naming of Treatment Plan Parameters

| CHARACTER NAME | SYMBOL |
| --- | --- |
| Backslash | \ |
| Equal | = |
| Caret | ^ |
| Period | . |
| Exclamation Mark | ! |
| At Sign | @ |
| Pound | # |
